# Small Cell Carcinoma of the Hypopharynx

**DOI:** 10.7759/cureus.2987

**Published:** 2018-07-16

**Authors:** Ruixiang Sun, Alysa Fairchild, Brock Debenham

**Affiliations:** 1 Faculty of Medicine and Dentistry, University of Alberta, Edmonton, CAN; 2 Radiation Oncology, Cross Cancer Institute, University of Alberta, Edmonton, CAN; 3 Radiation Oncology, Cross Cancer Center, University of Alberta, Edmonton, CAN

**Keywords:** hypopharynx, extrapulmonary small cell carcinoma, chemotherapy, radiation treatment, prophylactic cranial radiotherapy

## Abstract

Small cell carcinoma is rarely found to originate from the hypopharynx and there exists no treatment guidelines due to the small number of cases. Here, we present a case of a female patient with metastatic small cell carcinoma originating from the posterior hypopharynx with lymph node involvement. Her treatment consisted of chemotherapy with etoposide and cisplatin as well as radiation therapy. Her post-treatment computed tomography (CT) scan indicated resolution of the disease at the primary site and follow-up positron emission tomography (PET)-CT scan at three-month post radiation therapy revealed that the patient is clear of the disease.

## Introduction

Small cell carcinoma is an aggressive form of cancer that most commonly arise in the lungs, accounting for approximately 20% of all lung cancers [1]. Extrapulmonary manifestations of small cell carcinoma are rare, consisting of 2.5%-5% of all small cell carcinomas [2]. In the head and neck region, the larynx is the most common site and the hypopharynx is an extremely rare site for extrapulmonary small cell carcinoma [3]. Due to the small number of cases reported for small cell carcinoma of the hypopharynx, there exists no standards or guideline for treatment. Previous treatments include chemotherapy, radiation therapy, or a combination of chemotherapy and radiation. Primary surgical resection and neck dissection have in most cases failed for small cell carcinoma in the larynx and hypopharynx [4]. The median overall survival rate for laryngeal and hypopharyngeal small cell carcinoma was found to be 17.9 months and the median two-year survival rate was found to be 40.6% [5]. We present a case of primary small cell carcinoma of the hypopharynx in a 67-year-old female patient.

## Case presentation

**Figure 1:**
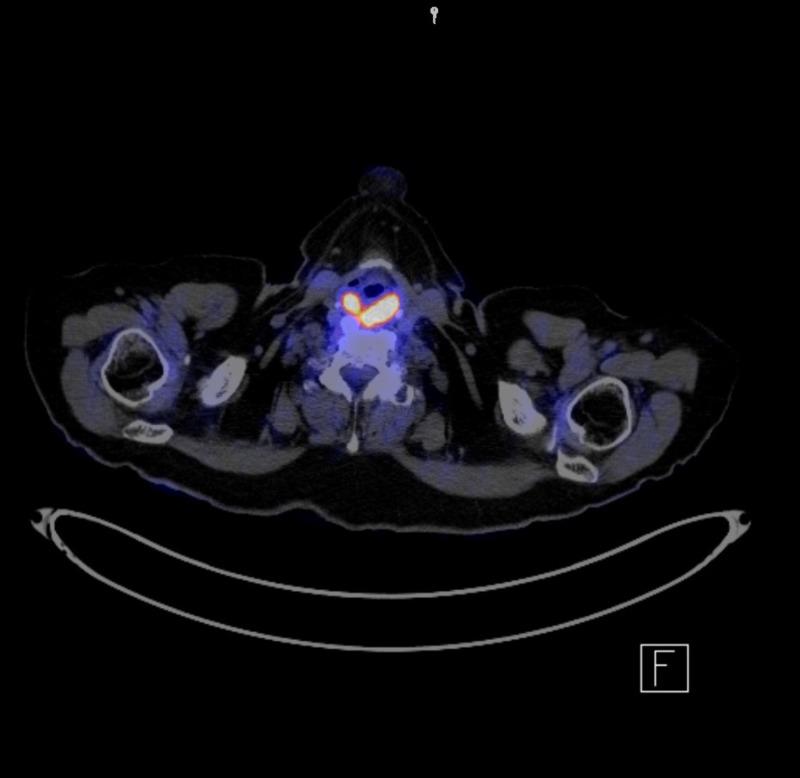
Pre-treatment positron emission tomography-computed tomography (PET/CT) scan

The patient presented to her family physician with a six-month history of a fluctuating neck mass. An ultrasound of the neck was obtained, which showed an abnormal lesion noted between the submandibular gland and the carotid vasculature suggestive of a grossly abnormal lymph node. A biopsy was obtained, which revealed metastatic small cell carcinoma. A chest X-ray was done, which was normal. A computed tomography (CT) scan of the head, chest, abdomen, and pelvis was done, which showed no evidence of disease in the chest or abdomen, but did show soft tissue fullness in the posterior hypopharynx. A positron emission tomography (PET)-CT scan was done, which showed prominent large left-sided level II lymph nodes measuring 3.9 x 2.1 cm with increased metabolic activity, as seen in Figure 1. The hypopharynx also was fluorodeoxyglucose(FDG)-avid, as well as a contralateral level II lymph node.

Past medical history was significant for hypertension, dyslipidemia, alcoholism, and hypothyroidism. She is a current smoker, with a previous 50 pack year smoking history.

She was started on cisplatin and etoposide chemotherapy. She began radiotherapy concurrently with her second cycle of chemotherapy. She received 60 Gy in 30 fractions to the gross disease and bilateral neck using a volumetric modulated arc therapy (VMAT) plan. She required placement of a percutaneous endoscopic gastrostomy (PEG) tube at the end of her treatment for nutritional support. She was planned for four cycles of chemotherapy, but only received three due to poor performance status. She did not receive prophylactic cranial irradiation (PCI).

**Figure 2:**
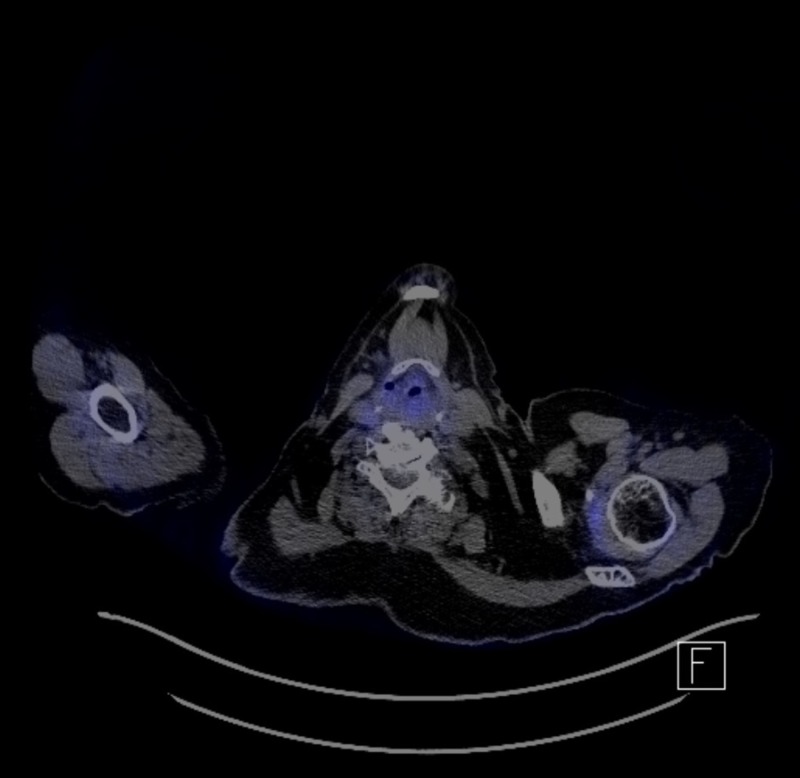
Post-treatment positron emission tomography-computed tomography (PET/CT) scan

Her initial post-treatment CT scan, done six weeks post treatment, demonstrated resolution of the primary site of disease, and a single remaining lymph node measuring 1 cm where it previously measured 3.9 cm. No distant disease was seen. CT head was normal. She had another PET/CT scan at three months post radiation therapy and was also clear of disease, as seen in Figure 2. 

. 

## Discussion

There have been cases reporting on hypopharyngeal small cell carcinoma and they are summarized in Table 1. Only cases reporting pure small cell carcinomas of the hypopharynx have been included. There are cases reporting combined small cell and squamous cell carcinomas of the hypopharynx; however, they have been excluded as the treatment guidelines for squamous cell carcinomas do not apply for small cell carcinomas.

**Table 1:** Cases of small cell carcinoma of the hypopharynx

Author	Year	Age/Sex	Smoking/Alcohol History	Metastasis	Treatment	Outcome
Lee et al. [3]	2016	71/M	50 pack-year smoking history. History of heavy alcohol use.	Hypermetabolic mediastinal lymph nodes found.	Chemotherapy: cisplatin and etoposide. Radiation: 70 Gy in 35 fractions.	Complete response to treatment; no evidence of recurrence over 11 months of follow up.
Bayram et al. [6]	2015	50/M	60 pack-year smoking history.	Metastatic nodules found in lungs.	Chemotherapy: cisplatin and etoposide. Radiation: 2.12 Gy/fraction/day until 16^th^ session.	After chemotherapy: complete response achieved at primary tumor site and the neck. The patient remained disease-free for 15 months.
Treglia et al. [7]	2014	64/M	Unknown	Lymph node and bone metastasis were found.	Chemotherapy only.	Died eight months later due to disease progression.
Gaba et al. [8]	2005	65/M	Excessive alcohol consumption. Remote tobacco history.	1.5 cm level III ipsilateral cervical lymph node. No pulmonary or distant metastasis	Laser debulking. Chemotherapy: platinum-based chemotherapy Radiation: 69 Gy of a planned hyperfractionated course of 74.4 Gy to the primary tumor.	Complete clinical response at 24 Gy. The patient remains disease-free two years after diagnosis.
Sano et al. [4]	2005	67/F	80 pack-year smoking history. History of alcohol use.	Right neck lymph node involvement.	Chemotherapy: carboplatin (CBDCA) and etoposide (VP-16) Radiation: 54 Gy (1.8 Gy x 30 fractions over seven weeks) delivered to the primary site, hypopharynx, and right neck lymph nodes.	Complete response was achieved with chemotherapy, but one month after last radiation, lung and liver metastasis appeared. Chemotherapy given but the patient died of lung and liver metastasis 13 months after initial diagnosis.
Yoshida et al. [1]	2005	78/M	50 pack-year smoking history. Moderate alcohol use.	No distant metastasis.	Chemotherapy: docetaxel, cisplatin, 5-FU Radiation: 2 Gy/day, five days/week. Total of 54 Gy over 27 fractions.	Since initial chemoradiotherapy, the patient has been free of disease for three years and there were no evidence of late complications of radiotherapy.
Baugh et al. [9]	1986	63/F	No tobacco or alcohol history.	No distant metastasis.	Surgical resection of largest neck mass. Chemotherapy: doxorubicin, Cytoxan, vincristine.	Right vocal cord remains paralyzed in the paramedian position. 55 months after initial diagnosis there were no evidence of recurrence of tumor.
Baugh et al. [9]	1986	35/M	No smoking history. Heavy Use of alcohol.	None.	Radiation: 66 Gy over 35 fractions delivered over 57 days to primary site and neck.	No evidence of disease 21 months after diagnosis.

Extrapulmonary small cell carcinomas are rare tumors that make up approximately 2.5-5% of small cell carcinomas [2]. They most frequently arise in the gastrointestinal and genitourinal tract, such as the esophagus and bladder [10]. For all sites in the head and neck region, the median survival is 20.3 months with a two-year overall survival of 45.2% [5]. Due to a low number of cases, treatment modalities for extrapulmonary small cell carcinomas have been based on case reports and series as well as treatment regimens for small cell carcinomas of the lung. It has been suggested that surgical resection and radiation be used for regional treatment while for relapses and metastasis chemotherapy regimens modelled after small cell carcinomas of the lung should be used [11]. For example, for early stages of small cell carcinoma of the bladder, transurethral resection of cystectomy is the initial treatment modality [11]. For extensive disease, the treatment regimen for small cell carcinoma, platinum-based chemotherapy with etoposide were found to prolong survival [10]. This was the chemotherapy regimen used in majority of case reports on hypopharngeal small cell carcinoma with favourable results, and was also used for the patient discussed in the report.

In the head and neck region, the most common site for extrapulmonary small cell carcinoma is the larynx [3,5]. Surgery is not recommended for primary treatment due to distant metastasis, local recurrence, as well as poor prognosis of the disease as surgical resection procedures such as total laryngectomy cause a significant decrease in quality of life [12]. The treatment for laryngeal small cell carcinoma with the best results is concurrent or sequential chemoradiotherapy commonly using platinum-based chemotherapy agents with etoposide [13]. This is similar to the chemotherapy regimen applied in most of the cases of hypopharyngeal small cell carcinoma presented here. Despite treatment, local and distant metastasis are common and prognosis are poor for laryngeal small cell carcinoma [13].

None of the pure small cell carcinomas of the hypopharynx cases reported gave PCI as part of the treatment plan. The literature remains controversial regarding giving PCI to patients with small cell carcinoma of the head and neck. Yacizi suggested that since extrapulmonary primary small cell carcinoma in the head and neck region have a high incidence of brain metastasis at 41%, PCI should be considered for these patients [14]. While Mason suggests that brain metastases are rare for these patients and prophylactic cranial irradiation is not warranted [15].

There are no guidelines for doses of radiation used to control the disease, although most cases indicate a high dose radical radiation dose of at least 50 Gy, except for palliative cases. This is similar for both laryngeal and hypopharyngeal small cell carcinoma [13]. Most of the cases reviewed employ radiation therapy in conjunction with chemotherapy.

Small cell carcinoma remains a cancer with relatively poor prognosis and due to a low incidence of extrapulmonary cases, especially those in the hypopharynx, large-scale studies and trials cannot be conducted. It would be beneficial to consider advancements in treatment modalities for small cell carcinomas of the lung, such as immunotherapy and apply them to extrapulmonary small cell carcinomas. Small cell lung cancer is closely related to abnormal regulation of autoimmunity, and promising advances have been made with immune checkpoint blockers [16].

## Conclusions

Small cell carcinoma is rarely found to originate from the hypopharynx. Due to the low number of cases, there are no guidelines available to guide treatment. Combined chemotherapy and radiation therapy was found to be a common treatment regimen as guided by treatment for small cell carcinoma of the lung.
